# Effect of Fatty Acid Polyunsaturation on Synthesis and Properties of Emulsion Polymers Based on Plant Oil-Based Acrylic Monomers

**DOI:** 10.3390/molecules27030932

**Published:** 2022-01-29

**Authors:** Vasylyna Kirianchuk, Zoriana Demchuk, Yehor Polunin, Ananiy Kohut, Stanislav Voronov, Andriy Voronov

**Affiliations:** 1 Department of Organic Chemistry, Institute of Chemistry and Chemical Technologies, Lviv Polytechnic National University, 79013 Lviv, Ukraine; vasuluna411@ukr.net (V.K.); ananiy.kohut@gmail.com (A.K.); stanislav.voronov@gmail.com (S.V.); 2 Department of Coatings and Polymeric Materials, North Dakota State University, Fargo, ND 58102, USA; zoriana.demchuk@ndsu.edu (Z.D.); yehor.polunin@ndsu.edu (Y.P.); 3 Oak Ridge National Laboratory, Oak Ridge, TN 37830, USA

**Keywords:** plant oils, plant oil-based acrylic monomers, emulsion polymerization, biobased latexes, crosslinked latex films

## Abstract

This study demonstrated that polymerization behavior of plant oil-based acrylic monomers (POBMs) synthesized in one-step transesterification reaction from naturally rich in oleic acid olive, canola, and high-oleic soybean oils is associated with a varying mass fraction of polyunsaturated fatty acid fragments (linoleic (C18:2) and linolenic (C18:3) acid esters) in plant oil. Using miniemulsion polymerization, a range of stable copolymer latexes was synthesized from 60 wt.% of each POBM and styrene to determine the impact of POBM chemical composition (polyunsaturation) on thermal and mechanical properties of the resulted polymeric materials. The unique composition of each plant oil serves as an experimental tool to determine the effect of polyunsaturated fatty acid fragments on POBM polymerization behavior and thermomechanical properties of crosslinked films made from POBM-based latexes. The obtained results show that increasing polyunsaturation in the copolymers results in an enhanced crosslink density of the latex polymer network which essentially impacts the mechanical properties of the films (both Young’s modulus and toughness). Maximum toughness was observed for crosslinked latex films made from 50 wt.% of each POBM in the monomer feed.

## 1. Introduction

Due to the depletion of fossil fuels and the environmental deterioration, there is a surge in research to find alternative feedstocks for polymer synthesis. Therefore, renewable raw materials, such as cellulose, lignin, vegetable oils, starches, and mono- and disaccharides, attract much attention in the synthesis of polymeric materials due to availability, low price, and rich application possibilities [1,2]. Owing to the abundance and high functionality, plant oils have become a prospective renewable feedstock for the synthesis of various biobased polymers and polymeric materials [3]. About 90% of chemical reactions using plant oil triglycerides occur through the ester group, whereas the rest of the transformations involve allyl fragments [4]. By using various methods, oxypolymerized oils, polyesters [5], polyurethanes [6], polyamides, acrylates, and epoxy resins based on plant oil triglycerides [7] were successfully developed.

An original one-step method of plant oil direct transesterification was recently reported [8,9] to synthesize plant oil-based acrylic monomers (POBMs). POBMs can undergo free-radical polymerization (including emulsion synthesis of biobased latexes) and, at the same time, uniquely retain reactive sites in fatty acid fragments to generate polymeric materials with advanced properties and extended performance in post-polymerization crosslinking. Depending on the oil(s) used, material properties can be tailored based on the fatty acid side chain content and unsaturation.

It is known that the oxidation rate of linoleic and linolenic acids, respectively, is 22 and 77 times higher than that of oleic acid [10]. As it has been demonstrated in our previous work, during the free-radical (co)polymerization of the POBMs, chain propagation and chain transfer (to the allylic C–H) cause the formation of more stable radicals and retardation of polymerization. This significantly affects the reaction rate and degree of (co)polymerization [9]. In this regard, in this study, we were focused on the synthesis and polymerizability of POBMs with a high content of oleic acid esters that can be of scientific and practical importance and has not been a research subject of our prior investigations.

In general, high-oleic plant oils, which have oleic acid content of over 70%, have attracted great attention due to higher oxidative and thermal stability. These oils are safe and suitable for use in both the food and non-food industries. Thus, olive, high-oleic soybean, high-oleic sunflower, high-oleic canola, and commodity canola oil experiences large-scale production and use in the world market of plant oils [11]. High-oleic soybean oil is a new and important monounsaturated plant oil that has enhanced functionality resulting in additional benefits to the food and industrial sectors. Canola oil (the name canola is a condensation of Canada and oil; low-acid) is a plant oil with less than 2% of monounsaturated erucic acid (C22:1) extracted from a genetically modified rapeseed which is the main difference between canola and commodity rapeseed oil.

Similar to olive oil, canola oil possesses a substantial amount of monounsaturated fatty acids, but unlike olive acid, it is rich in omega-3 fatty acids. Evidently, the synthesis of monomers based on canola and high-oleic soybean oil containing a high amount of oleic acid esters may extend the range of the POBM applications. Moreover, a high content of oleic acid esters (C18:1) can allow for a higher total monomer conversion along with the less-pronounced retardation during the polymerization in comparison with more unsaturated POBMs [8]. This may ensure the broader range of homo- and copolymers in terms of molecular weight as well as help to investigate and better understand the effect of polyunsaturated fragments on the properties and performance of POBM-based polymeric materials.

The current literature overview indicates that although mechanical properties of polymeric materials based on plant-oil-derived ingredients are determined by the varying unsaturation degree in the macromolecules [12], the specific effects of unsaturated fatty acid fragments on synthesis, characteristics, and properties of crosslinked biobased latex films remain mainly unexplored.

Herein, in this study, we focused on revealing the effect of polyunsaturated fatty acid fragments in plant oils on the POBM polymerization behavior as well as on the mechanical performance of POBM-based crosslinked latex films. For this purpose, three oils with a similar content of oleic acid esters and a widely different polyunsaturated content, such as olive, high-oleic soybean, and canola oil, were chosen for the POBM synthesis. Our goal was to examine the impact of linoleic (C18:2) and linolenic (C18:3) acid content in the fatty acid profiles of the chosen for monomer synthesis plant oil (and, respectively, in POBMs) on: i. POBM polymerization kinetics and ii. the thermomechanical properties of crosslinked POBM-based latex films with a varying content of linoleic and linolenic acids.

Latexes with a high biobased content (40–60 wt.%) of monomers from olive oil (OVM), high-oleic soybean oil (HOSBM), and canola oil (CLM) copolymerized in a miniemulsion process with styrene were synthesized. We hypothesized that the mass fraction of each polyunsaturated fragment type in the plant oil can be utilized as a criterion to determine polyunsaturation effects on both the resulting latex characteristics and thermomechanical properties of crosslinked latex films.

## 2. Results and Discussion

### 2.1. Synthesis, Characterization, and Polymerization of OVM, CLM, and HOSBM

To synthesize acrylic monomers with a high content of oleic acid esters, commercially available olive, canola, and high-oleic soybean oils were chosen. The fatty acid composition of plant oils depends on the source plant, environmental conditions during plant growth, the technology process for oil production, and so forth. Thus, the composition of the plant oils chosen for monomer synthesis was determined at first using ^1^H NMR spectroscopy according to the approach reported in [13]. The ^1^H NMR spectra of plant oils used in this study are shown in Figure S1 in the Supporting Information.

**Figure 1:**
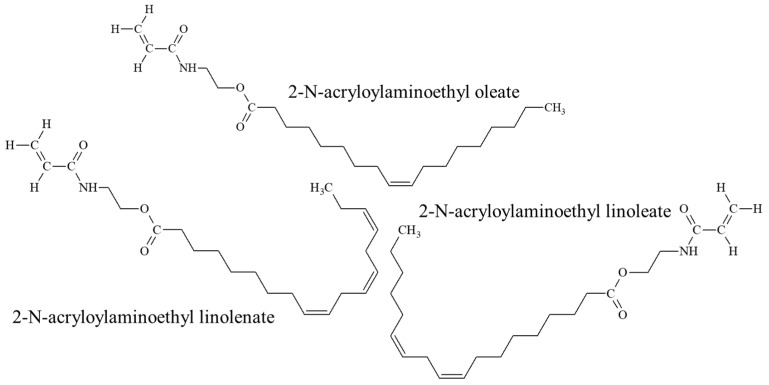
Chemical structure of plant-oil-based monomer mixtures.

The chosen oils’ composition differs in linoleic (C18:2) and linolenic (C18:3) acid content (Table 1), which makes polyunsaturation amount an important experimental parameter for the study of POBM polymerizability. As demonstrated in our previous work, the chemical composition of oil triglycerides remains unaltered during POBM synthesis and, therefore, entirely determines the chemical composition of a corresponding POBM [14]. Hence, upon the transesterification, POBMs predominantly consist of 2-N-acryloylaminoethyl oleate with various fractions of 2-N-acryloylaminoethyl linoleate and 2-N-acryloylaminoethyl linolenate as admixtures were formed in this work (Figure 1).

Physico-chemical characteristics (namely, the iodine value, density, and refractive index) were determined for each synthesized monomer (Table 1). To determine the degree of unsaturation of the prepared POBMs, the iodine value was measured (amount of iodine in g that reacts with fatty acid chain double bonds of 100 g of a substance at specified conditions) and compared to the iodine value of the corresponding oil chosen for monomer synthesis. The iodine value for all POBMs is larger than for oils, confirming the incorporation of the vinyl group in the POBM structure during monomer synthesis (Table 1). The iodine value increases in the range OVM < HOSBM < CLM which is determined by increasing polyunsaturation in this monomer range (Table 1). The aqueous solubility of the POBMs was determined using UV−vis spectrometry by measuring the cloud point/transparency of diluted monomer solutions in distilled water. Due to their highly hydrophobic nature, the POBMs show very limited aqueous solubility (0.9–1.05 · 10^−3^ %). The density values of OVM, HOSBM, and CLM are found to be somewhat higher than those of corresponding crude plant oil owing to the introduction of an acryloylamide moiety into the monomer structure.

**Figure 2:**
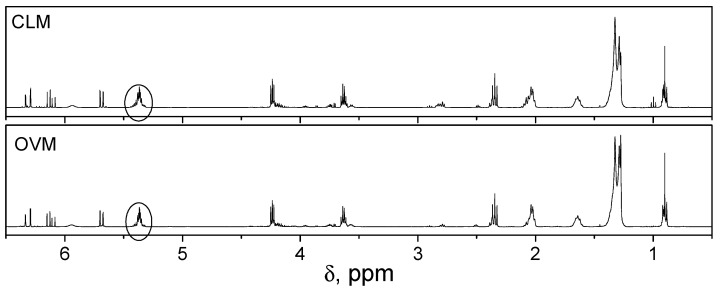
^1^H NMR spectra of the CLM and OVM.

The chemical structure of the synthesized POBMs was confirmed using ^1^H NMR spectroscopy (Figure 2). The spectra show the presence of the proton signals at 6–6.6 ppm (3H for both CLM and OVM) that are typical for an acrylic carbon–carbon double bond, the peaks of the two methylene group protons located between the amide and ester groups at 3.6 and 4.20 ppm, respectively (2H and 2H for both monomers), as well as the peaks of the protons of the fatty acid chains (from 0.8 to 2.8 ppm).

**Figure 3:**
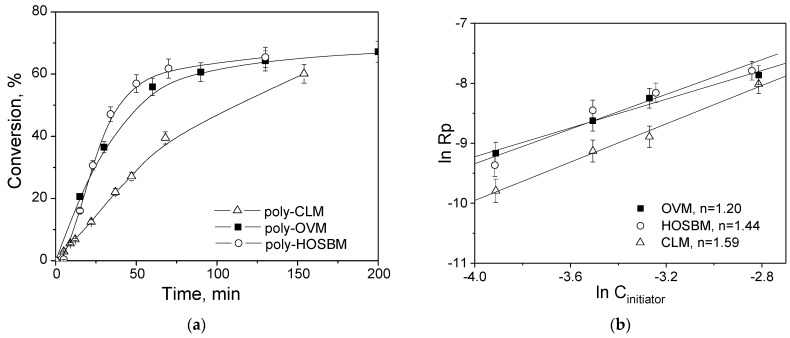
Conversion–time change in polymerization of POBMs (**a**) and polymerization rate of the POBMs vs. initiator concentration at 75 °C (**b**).

Having both the fatty acid profiles of the plant oils and the confirmed structure of the synthesized POBMs, we studied how the difference in linoleic (C18:2) and linolenic (C18:3) acid fragment content impacts the polymerization kinetics. To this end, the features of the free-radical polymerization of OVM, CLM, and HOSBM were studied. According to the composition of the POBMs (Table 1), the influence of linoleic (C18:2) and linolenic (C18:3) acid content can be investigated via comparison of the polymerization behavior for OVM vs. HOSBM (where the C18:2 and C18:3 contents are about twice as different), as well as for HOSBM vs. CLM (where the content of C18:3 is sixfold higher for CLM). Comparing the conversion–time dependence in the polymerization of HOSBM and OVM (Figure 3a), it is clearly seen that the conversion values in both reactions are similar within 2 h, while for CLM (which is significantly more polyunsaturated), a lower polymerization rate is observed. Thus, it can be assumed that the presence of polyunsaturated fragments in monomer feed predominantly affects the rate of POBM polymerization.

Table 2 provides the characteristics of the polymerization of 1 M of monomer initiated by 0.038 M of the initiator AIBN at 75 °C. It is evident that both the reaction rate and the number average molecular weight of the homopolymers decrease with the increasing content of the polyunsaturated fatty acid fragments in OVM (7.3%) > HOSBM (16.5%) > CLM (28.5%).

It is well known that the hydrogen atoms in methylene groups located between double bonds (presented in the polyunsaturated acid fragments of POBMs, Figure 1) can undergo chain transfer reactions and act as retarders of polymerization which explains the diminishing of reaction rate from OVM to CLM [15]. To further elaborate the kinetic features of polymerization and evaluate the impact of POBM polyunsaturation on chain transfer, the reaction order with respect to the initiator was determined in the polymerization of 1 M of each POBM initiated by different concentrations of AIBN at 75 °C (Figure 3b).

For each POBM polymerization, a significant deviation of a reaction order with respect to the initiator from the free-radical polymerization conventional kinetics (when reaction order value 0.5 is expected) is found. The higher values for all three monomers are explained by the presence of polyunsaturated fragments in the POBM chemical structure, most pronounced for CLM which contains a greater number of allylic hydrogens prone to chain transfer in its molecule [9].

Although the order of polymerization reaction with respect to the initiator increases with increasing POBM polyunsaturation, the number of the protons of the carbon−carbon double bonds in the fatty acid chains almost did not change significantly after polymerization (Figure 2) (the proton signal at 5.37 ppm; 1.97 H in poly(OVM) vs. 1.98 H in OVM and 2.91 H in poly(CLM) vs. 2.86 H in CLM). This indicates that only 0.5–2% of such bonds take part in allylic chain transfer; thus, their vast majority remains intact and available for post-polymerization reactions.

To quantify the effect of polyunsaturated POBM fragments on polymerization kinetics, a chain transfer constant (C_M_) was determined for each POBM. The obtained values clearly correspond to the POBM chemical structure with respect to varying polyunsaturation content (number of C-H groups in the α-position of the fatty acid double bonds in POBM macromolecules)—0.025(CLM) > 0.018 (HOSBM) > 0.015 (OVM) which indicates the most-expressed retardation due to the allylic chain transfer during polymerization of CLM which has the highest in this range polyunsaturated content.

Furthermore, the specific effect of linolenic acid moieties (C18:3) in POBMs was identified by comparing the obtained data for CLM with those obtained in our previous study of soybean oil-based monomer (SBM) [8,9] that has a similar total unsaturation extent (indicated by similar IV value, 137 for CLM and 141 for SBM) but varies in terms of polyunsaturation. In particular, the content of C18:1 and C18:2 in both CLM and SBM differs significantly, whereas the content of C18:3 is close (7.3% for CLM and 10% for SBM). We hypothesized that since most obtained kinetics characteristics are similar for polymerizations of CLM and SBM (including reaction order to initiator—1.44 and 1.53; chain transfer constant—0.025 and 0.026, respectively; number average molecular weight—about 14,200 for both homopolymers), the prevailing specific impact of linoleic acid fragments on both POBM kinetics and polymer molecular weight can be assumed. Thus, this similarity for SBM and CLM of C18:3 determines polymerization behavior while the contribution of two other fractions appears to be less influential.

### 2.2. Impact of POBM Unsaturation on Properties of Latex Copolymers and Films Thereof

A wide variety of specialty materials can be manufactured using emulsion (stable liquid–liquid dispersion) [16]. Free-radical polymerization in emulsion is the process applied for producing latexes and advanced polymeric materials and is widely used for making coatings, paints, adhesives, etc. One of the major factors affecting the properties and performance of latex is its formulation and chemical composition [17]. As we already demonstrated, the presence of plant-oil-based unsaturated fragments in latex copolymers provides an experimental tool to crosslink resulting latexes in a controlled way and advance the polymeric materials’ thermomechanical properties and performance [18].

Fundamental latex properties, including total monomer conversion, latex particle size distribution, copolymer composition, and molecular weight are directly related to this material performance and its intended application [17].

In this study, to reveal the overall effect of fatty acid unsaturation in plant oil, first, we examined the effect of POBM chemical composition on properties of the latex film made from a high concentration of each plant oil-based monomer in reaction feed. For this purpose, four latex copolymers based on 60% by weight of POBMs with a different extent of total unsaturation were synthesized. We hypothesized that incorporation of this high amount of POBMs with a different unsaturation extent (IV:110–141 g/g) in monomer feed provides robust background information to further investigate structure–property relationships of POBM-based latexes and latex films. The obtained results (Table 3) indicate that, as expected, the number average molecular weight (Mn) of the latex copolymers decreases with an increasing mass fraction of polyunsaturated fatty acid fragments in the resulted materials (W_PUF_). The latter observation shows that the molecular weight of latexes made from a high amount of POBMs can be controlled by varying the polyunsaturation in the plant oil chosen for monomer synthesis. Furthermore, DSC measurements confirm (Table 3) that the variation of polyunsaturation in the oil also affects the thermal properties of the resulted highly biobased latexes by decreasing the glass transition temperature and internal plasticization effect of POBM-based fragments [19]. Most double bonds in POBM fatty fragments are retained during polymerization (Figure 2) and can be used for the crosslinking of latex films and coatings to form polymer networks [9]. As the obtained data show (Table 3), a higher POBM content in the feed results in an increased crosslink density of the materials causing the noticeable variation effect on the glass transition temperature changing in a range of 4.7–27 °C (for uncured latexes copolymers) to 40–52.9 °C (for cured latex films).

Taking into account all the observations (Table 3), our next step in this study was to specifically consider the effect of polyunsaturated—C18:2 linoleic acid- and C18: 3 linolenic acid-based fractions of each POBM on the properties of highly biobased latexes and their crosslinked films’ mechanical performance.

For this purpose, a range of stable latexes with a varying biobased content was synthesized in miniemulsion from OVM, HOSBM, and CLM copolymerized with styrene in the presence of 4% surfactant (SDS, based on oil ph.). The latex solid content was kept at 30 wt.% for all polymerizations. Total monomer conversion obtained after 5 h of polymerization was determined by multiple precipitations of latex copolymers in methanol. The purified copolymers were dried in an oven until constant weight. The total monomer conversion values of 80–89% were obtained (Table 4). The resulted latex particle size distribution is provided in Figure S2 in the Supporting Information. The latexes were stable at room temperature for at least 6 months. The POBM-based latex copolymer composition was determined using ^1^H NMR spectroscopy. The obtained results indicate that a major fraction of POBMs is incorporated into the latex copolymers during polymerization (Table 4). As expected, the molecular weight of latex copolymers decreases with increasing the W_PUF_ corresponding to each monomeric pair.

To evaluate the effect of polyunsaturation on latex film properties, the mass fraction of unsaturated fragments was calculated and is provided in Table 4 for each synthesized latex. To form highly biobased crosslinked films, POBM-based latexes were cast on a glass substrate and cured at elevated temperature for 6 h. The resulted films were peeled off from the substrate and tested using DMA and tensile testing (to determine tensile strength, elongation at break ε_br_, Young’s modulus E, toughness, crosslink density (*XLD*), etc.).

The obtained results (Table 5) indicate that the Tg and *XLD* of biobased latex films increase when W_PUF_ increases if different amounts of POBMs made from plant oils with various polyunsaturation are used in miniemulsion polymerization. The data also show that the tensile properties of highly biobased latex films are determined by *XLD* and thus depend on the resulted crosslinked copolymer structure. A comparison of Young’s modulus and tensile strength values of the crosslinked latex films shows that both E and σ increase in the range: OVM < HOSBM < CLM. This can be explained by increasing polyunsaturation content in this range which significantly affects the macromolecular length (as it is shown in Table 3 and Table 4, molecular weight of latex copolymers is typically higher at lower W_PUF_), and, respectively, *XLD*, thus leading to lower elastic deformation.

Due to the higher amount of double bonds available for crosslinking reactions via auto-oxidation, a higher polyunsaturation content causes a rise in the *XLD* of crosslinked latex films, observed for POBM varying content. The most apparent change in Young′s modulus (E), tensile strength (σ), and elongation at break is observed when the POBM content increases from 40% to 50%. It is worth pointing out that copolymers from 40 wt.% of HOSBM and CLM contain similar mass fractions of C18:1 and C18:2, while the C18:3 amount in CLM is higher more than five times. The latter characteristics can be utilized to distinguish the effects of polyunsaturated fragments on the thermal and mechanical properties of crosslinked latex films. Increasing the content of C18:3 in the latexes synthesized from 40 wt.% of CLM in comparison with the material from the same concentration of HOSBM results in a noticeable increase of both Young′s modulus and tensile strength values, as well as a decrease in the elongation at break. This can be explained by a considerably higher *XLD* of latex films from CLM-based copolymer (0.97 mol/cm^3^ compared to 0.5–0.56 mol/cm^3^ for OVM- and HOSBM-based copolymers, respectively) caused by the greater content of C18:3. When biobased content is 50 wt.% (and higher) (Table 5), the mechanical properties of the crosslinked latex films are mainly determined by W_PUF_ while the specific effect of C18:3 becomes diminished.

Another new and strategic observation made from data in Table 5 is a significant increase of elongation at break when POBM content in monomer feed increases from 40 to 50 wt.%, while the tensile strength of the tested materials remains essentially unchanged. We attribute the increased flexibility to configurational changes (presence of entanglements of dangling macromolecular side fragments) occurring at high POBM content in copolymer composition [20]. Those entanglements can stretch and pull apart, thus triggering greater flexibility and toughness. This effect is less pronounced for the polymers with a lower degree of unsaturation since such macromolecules are longer and have fewer entanglements per macromolecule. As the degree of POBM polyunsaturation grows (OVM < HOSBM < CLM), the resulted synthesized macromolecules become shorter, thus impacted by more populated entanglements due to more densely distributed side fragments.

**Figure 4:**
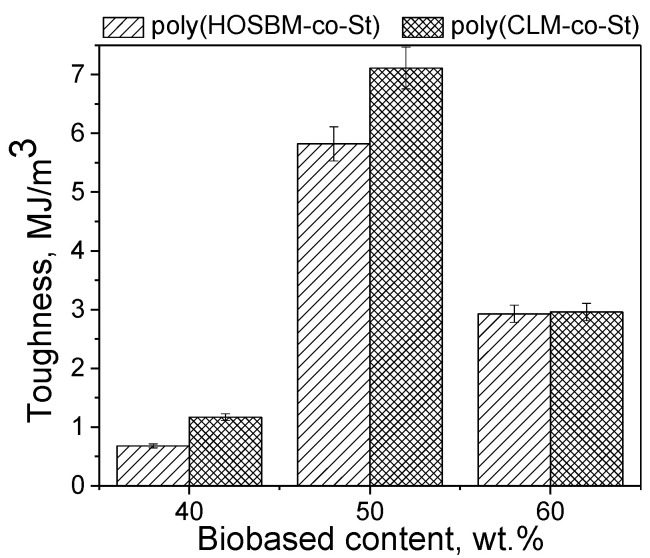
Effect of the POBM composition and content on the toughness of crosslinked latex films.

Figure 4 shows the effect of the POBM composition and content in latex copolymers on the toughness values of the crosslinked latex films. By increasing biobased content from 40 to 50 wt.%, toughness values increase sharply while for copolymers with 60 wt.%, a POBM rapid drop in toughness is observed. We presume that this sharp increase in toughness can be assigned to the presence of the entanglements of fatty acid side chains in macromolecules as we discussed above. When POBM fragments are incorporated into latex copolymers at a higher extent (60 wt.%), the crosslinking density becomes excessive, causing loss of the resulted polymer network flexibility and stiffening the resulted films made from copolymers based on both HOSBM and CLM.

To provide a more quantitative evaluation of how the extent of POBM polyunsaturation impacts the *XLD* (and, correspondingly, crosslinked latex films properties), the monomer average number of double bonds (*ADB*) [21] and the factor of unsaturation (*FU*) were calculated using the following equations:

(1) $$ADB=\frac{\sum \left(FA_{i}\cdot n_{DB,i}\right)}{\sum FA_{i}}$$

(2) $$FU=C_{m}\cdot ADB$$

where *n_DB,i_*—number of double bonds of the fatty acid; *FA_i_*—mass fraction of the fatty acid *i*, taken from the fatty acid monomer composition; *Cm*—content of monomeric fatty acid fragments in the copolymer composition, determined using ^1^H NMR spectroscopy.

The calculated *ADB* values increase with the increasing polyunsaturated content of POBMs in the range: OVM (0.955) < HOSBM (1.044) < CLM (1.303).

**Figure 5:**
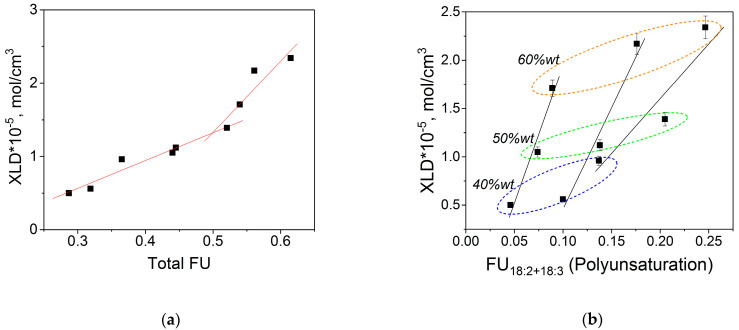
Plots of the crosslink density of the biobased polymer network vs. total *FU* (**a**) and vs. polyunsaturation (**b**).

Figure 5a shows the effect of the total unsaturation of latexes, *FU*, which is a sum of *FU*_18:1_, *FU*_18:2_, and *FU*_18:3_ in each synthesized copolymer, on the *XLD* of the POBM-based latex films. It is evident that at a certain value of 0.52 (corresponding to the copolymer from 50 wt.% CLM), the *XLD* increases sharply. This effect can be explained by the presence of fatty acid double bonds in macromolecules in amounts sufficient to be located in film morphology close to each other, thus, facilitating more pronounced and extensive crosslinking [22]. The obtained results indicate that the latter effect occurs when the *FU* of biobased latex copolymers reaches the range of 0.5–0.6, resulting in a considerable enhancement of the *XLD* from 1.39 to 2.34 mol/cm^3^. This is clearly illustrated in Figure 5a indicating that for all three copolymers made of 60% wt. POBM, the *XLD* increases abruptly, and much stiffer polymer networks are formed after crosslinking (Table 5**)**.

Figure 5b shows how combined *FU*_18:2_ and *FU*_18:3_ (polyunsaturation) impact the *XLD* of crosslinked latex films. The polyunsaturation effect is clearly most pronounced for latexes made of 60 wt.% POBMs. The latter is coherent with the experimentally observed toughness values drop (Figure 4) and can be explained by formation of more rigid latex polymer networks at this high concentration of incorporated POBM fragments in latex copolymers. Data in Figure 5b also show that varying the ratio of monounsaturated (C18:1): polyunsaturated (C18:2 + C18:3) fragments in monomer feed provides a versatile tool for controlling crosslinked latex films’ thermomechanical properties.

## 3. Materials and Methods

### 3.1. Materials

Olive oil (Bertolli; Houston, TX, USA), high-oleic soybean oil (Perdue Agribusiness LLC, Salisbury, MD, USA), canola oil (SuperValu Inc., Eden Prairie, MN, USA), and N-(hydroxyethyl)acrylamide (HEAAm; TCI America, Portland, OR, USA) were used as received. Azobis(isobutyronitrile) (AIBN; Sigma-Aldrich, St. Louis, MO, USA) was purified with recrystallization from methanol. Toluene (Sigma-Aldrich, St. Louis, MO, USA) was distilled prior to use. Other solvents and chemicals, all analytical grade or better, were used as received.

### 3.2. Monomer Synthesis

Acrylic monomers from plant oils were synthesized using one-step transesterification reaction of N-hydroxyethyl acrylamide with plant oil triglycerides in the presence of catalytic amounts of sodium hydroxide. The detailed synthesis procedure is described in [8,9]. The monomer yield was about 92–96% in each synthesis.

### 3.3. Solution Polymerization of POBMs

To prepare POBM-based homopolymers, free-radical polymerization of each plant oil-based monomer (1.0 M) was conducted in toluene using AIBN (0.038 M) as initiator according to technique [9]. The resulting POBM-based homopolymers were purified by multiple precipitations from toluene in methanol and dried at room temperature until a constant weight was obtained.

### 3.4. Kinetic Study of POBM Polymerization

The study was carried out gravimetrically by precipitation of a fraction of the reaction mixture in methanol. The obtained product was centrifuged in a weighted tube at 4000 rpm for 5–10 min to ensure complete precipitation of the polymer. The supernatant was poured out, and the precipitate was dried in vacuum until a constant weight was obtained.

To establish the order of reaction with respect to initiator, polymerization was conducted at a constant monomer concentration (C_monomer_ = 1.0 M) and varied AIBN initiator concentrations (C_initiator_ = 0.02–0.06 M) at 75 °C. For each initiator concentration, the polymerization rate (Rp, mol L^−1^ s^−1^) was determined on a linear part of the conversion vs. time plot (up to a 10–15% monomer conversion). Then, ln Rp was plotted against ln (C_initiator_), and a slope of the straight line (i.e., tan α which corresponds to the order of reaction with respect to initiator) was calculated [15].

To evaluate the extent of the effect of chain transfer on polymerization, the established Mayo method [15] was employed to determine the values of chain transfer constants on monomer (C_M_, ratio of the chain transfer and propagation rate constants) in polymerization of plant-oil-based monomers at 75 °C. In this method, the inverse value of the number average degree of polymerization is plotted against the polymerization rate, and C_M_ is determined by the intercept by extrapolating to zero rate.

### 3.5. POBMs and POBM-Based Polymer Characterization

The chemical structure of the plant oils, POBMs, and POBM-based polymers was confirmed by ^1^H NMR spectroscopy (AVANCE III HDTM 400 high-performance digital NMR spectrometer, Bruker, Billerica, MA, USA) using CDCl_3_ as a solvent. The aqueous solubility of the POBMs was determined using UV−vis spectrometry. The refractive index of the POBMs was measured using a Bausch & Lomb Refractometer. The density of the plant oils and the monomers was determined according to the standard procedure [9]. The average molecular weight of the POBM-based homopolymer was determined by gel permeation chromatography (GPC) using a Waters Corporation modular chromatograph consisting of a Waters 515 HPLC pump, Waters 2410 Refractive Index Detector, and a set of two 10 μm PL-gel mixed-B columns; the column temperature was set at 40 °C. Tetrahydrofuran (THF) was used as the carrier solvent.

### 3.6. Latex Synthesis

Acrylic latexes were synthesized in miniemulsion copolymerization of respective POBM with St. For this purpose, oil phase (15 g) was prepared by mixing of each plant-oil-based monomer (40–60 wt.%, 6–9 g) with St at different ratios (6–9 g) in the presence of 1.5 wt.% (0.225 g) oil-soluble initiator. The detailed latex synthesis procedure and its characterization are described in [19].

### 3.7. Plant Oil-Based Latex Characterization

The latex solid content was measured gravimetrically by drying the latex samples in an oven at elevated temperature for 45 min.

Particle size distribution of the plant oil-based latex particles was measured using dynamic light scattering (Particle Sizing Systems Nicomp 380, Santa Barbara, CA, USA) at a scattering angle of 90 °C at room temperature.

The glass transition temperature of POBM-based latexes was determined by differential scanning calorimetry (DSC) (TA Instruments Q1000 calorimeter) at heat/cool/heat mode (−50 °C/150 °C) with dry nitrogen purging through the sample at 50 mL/min flow rate. The heating/cooling rate of latex samples was 10–20 °C/min.

Latex-free films were formed by applying latex on the clean glass substrate using a drawdown bar of 8 mils of thickness and curing at elevated temperature for 4–6 h to ensure autoxidation. The resulting films were peeled off from the substrate after curing.

The latex films were subjected to dynamic mechanical analysis (DMA; TA Instruments Q800) with a heating rate of 5 °C/min to investigate the dynamic mechanical behavior of plant oil-based latex films. The crosslink density of the latex films (*XLD*) was calculated using rubber elasticity theory [18]:

(3) XLD=G′(R·T) 

where *G*′—storing modulus at rubbery plateau region at T temperature; *R*—gas constant; *T*—temperature at rubbery plateau region (Tg + 60 °C).

Mechanical properties of latex films were tested using Instron tensile testing machine 2710-004 (Instron, Norwood, MA, US) with the maximum load of 500 N. The film samples had a rectangular shape (length: 25 mm, width: 5 mm). The thickness of the films was measured before each testing.

## 4. Conclusions

Plant oil-based acrylic monomers (POBMs), rich in oleic acid fragments, were synthesized via transesterification of olive, canola, and high-oleic soybean oil. Both polymerization kinetics of POBMs and the resulted polymer characteristics appear to be affected by polyunsaturated fractions (linoleic C18:2 and linolenic C18:3) of POBMs.

To determine the impact of POBM polyunsaturation on the thermomechanical properties of the resulted polymers, latexes based on 40–60 wt.% of each monomer copolymerized in a miniemulsion process with styrene were synthesized. For the crosslinked latex films, the increasing E and σ were observed in the range olive oil-based monomer (OVM) < high-oleic soybean oil-based monomer (HOSBM) < canola oil-based monomer (CLM) corresponding to increasing polyunsaturation in the resulted macromolecules and were determined by the most polyunsaturated fragments of linolenic acid (C18:3). At high biobased content in the copolymers (50 wt.% and above), the effect of C18:3 diminishes, and the mechanical properties of the crosslinked latex films depend mainly on total polyunsaturation.

In terms of the mechanical properties of crosslinked films made from highly biobased latexes, increasing POBM content from 40 to 50 wt.% leads to a sharp increase of toughness values, while much less flexible films are formed from copolymers based on 60 wt.% POBM. We relate this finding to an excessive *XLD* of latex films, enhancing the stiffness of the crosslinked materials.

Overall, the obtained results show that varying the ratio of monounsaturated (C18:1): polyunsaturated (C18:2 + C18:3) fragments in POBM-based monomeric feed provides a versatile tool for controlling thermomechanical properties of crosslinked latex films. The latter is demonstrated by the linear dependence of latex films’ crosslinked density on the calculated factor of polyunsaturation for each POBM content and each monomer investigated in this study.

## 5. Patents

Biobased Acrylic Monomers US 10,315,985 B2 11 June 2019.

Biobased Acrylic Monomers and Polymers Thereof US 10,584,094 B2 10 March 2020.

## Figures and Tables

**Table 1:** Physico-chemical characteristics of the POBMs.

Monomer	Fatty Acid Composition of Oil, wt.%				IV (Oil), g/100 g	Density (Oil), g/cm^3^	n_D_^20^ (Oil)
	C_18:1_	C_18:2_	C_18:3_	Other			
OVM	80.2	6.6	0.7	12.4	110 (90)	0.953 (0.912)	1.473 (1.469)
HOSBM	70.2	15.3	1.2	13.3	124 (105)	0.945 (0.910)	1.475 (1.469)
CLM	66.0	21.2	7.3	5.5	137 (111)	0.957 (0.914)	1.474 (1.470)

**Table 2:** Rate of polymerization, number average molecular weight, and polydispersity index of the POBM homopolymers.

Monomer	Rp·10^5^, mol L^−1^ s^−1^	Mn, g/mol	PDI	Polyunsaturated Fatty Acid Content, wt.%
OVM	26.1	21,200	1.37	7.3
HOSBM	17.1	18,000	1.40	16.5
CLM	13.7	14,200	1.42	28.5

**Table 3:** The effect of unsaturation on POBM latex properties.

Biobased Content (Feed), wt.%	Biobased Content (pol), wt.%	W_PUF_, wt.%	Mn, g/mol	Tg (pol), °C	Tg (Film), °C	Gel Content, %	*XLD*·10⁴, mol/cm³
60 OVM	56.5	4.1	108,500	18	52.9	71.0	1.71
60 HOSBM	53.8	8.9	110,700	22	40.6	70.0	2.17
60 CLM	50.0	14.3	66,400	27	48.0	81.0	2.34
60 SBM	56.1	29.1	39,100	4.7	43.7	80.6	2.61

**Table 4:** Characteristics of latexes from POBM and St.

Biobased Content, wt.%			Conversion, %	Molecular Weight Mn, g/mol	PDI	Mass Fraction of Unsaturated Acids, wt.%		
Feed		Polymer				C18:1	C18:2	C18:3
**40**	**OVM**	30.1	83	168,000	3.1	24.1	1.99	0.21
**50**		46.0	82	117,000	4.6	36.9	3.04	0.32
**60**		56.5	80	108,500	3.2	45.3	3.73	0.40
**40**	**HOSBM**	30.6	89	152,200	5.2	21.5	4.68	0.37
**50**		42.5	86	115,900	4.4	29.8	6.50	0.51
**60**		53.8	83	110,700	5.3	37.8	8.23	0.65
**40**	**CLM**	28.0	87	112,000	5.5	18.5	5.94	2.04
**50**		41.5	85	78,200	5.4	27.4	8.80	3.03
**60**		50.0	81	66,400	5.3	33.0	10.60	3.65

**Table 5:** Tensile properties of latex films from POBM copolymerized with St.

Biobased Content, wt.%	W_PUF_, wt.%	Tg (Film), °C	*XLD*·10⁴, mol/cm³	E, MPa	σ, MPa	ε_br_, %
40 OVM	2.2	67.1	0.50	290.0	4.4	24
40 HOSBM	5.1	70.7	0.56	342.0	4.3	20
40 CLM	8.0	76.0	0.96	475.0	11.9	14
50 OVM	3.4	57.1	1.05	92.0	3.3	245
50 HOSBM	7.0	58.2	1.12	64.1	3.3	236
50 CLM	11.8	65.0	1.39	250.0	4.2	175
60 OVM	4.1	52.9	1.71	2.1	2.3	275
60 HOSBM	8.9	48.1	2.17	11.9	2.2	270
60 CLM	14.3	48.0	2.34	95.0	2.7	155

## Data Availability

Data are contained within the article.

## Supplementary Materials

The following are available online, Figure S1. 1H NMR spectra of canola and olive oils, Figure S2. Latex particle size distribution POBM-St 60–40 wt.%.
