# Supplementary material for: Effect of Fatty Acid Polyunsaturation on Synthesis and Properties of Emulsion Polymers Based on Plant Oil-Based Acrylic Monomers
Source: Molecules. 2022 Jan 29;27(3):932. doi: 10.3390/molecules27030932 (PMC8839316; doi:10.3390/molecules27030932)
Supplement: Supplementary file 1 [file molecules-27-00932-s001.zip › molecules-1534123-supplementary.pdf]

Supporting Information

Effect of Fatty Acid Polyunsaturation on Synthesis and Properties of Emulsion Polymers Based on Plant Oil-Based Acrylic Monomers

Vasylyna Kirianchuk [1], Zoriana Demchuk [2,3], Yehor Polunin [2], Ananiy Kohut [1], Stanislav Voronov [1,†] and Andriy Voronov [2,*]

[1] Department of Organic Chemistry, Institute of Chemistry and Chemical Technologies, Lviv Polytechnic National University, 79013 Lviv, Ukraine; vasuluna411@ukr.net (V.K.); ananiy.kohut@gmail.com (A.K.); stanislav.voronov@gmail.com (S.V.)
[2] Department of Coatings and Polymeric Materials, North Dakota State University, Fargo, ND 58102, USA; zoriana.demchuk@ndsu.edu (Z.D.); yehor.polunin@ndsu.edu (Y.P.)
[3] Oak Ridge National Laboratory, Oak Ridge, TN 37830, USA
* Correspondence: andriy.voronov@ndsu.edu
† Author is deceased, 18 July 2021.

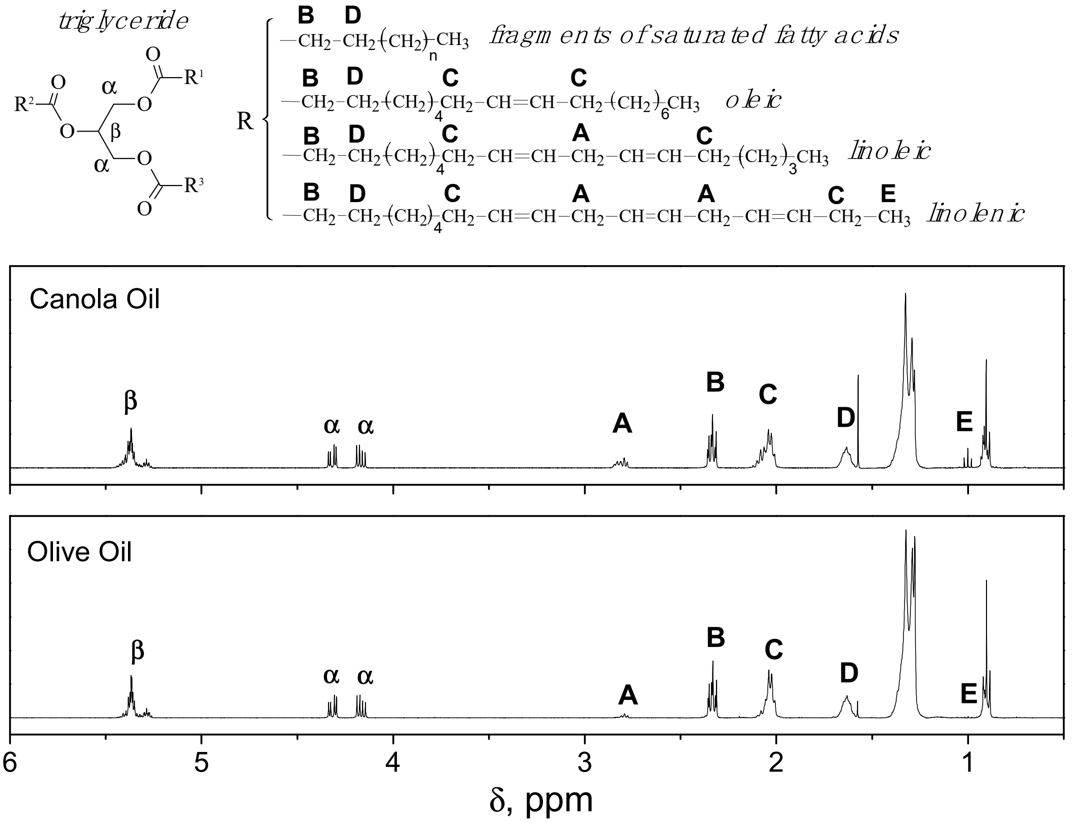

Figure S1. ^{1}H NMR spectra of canola and olive oils.

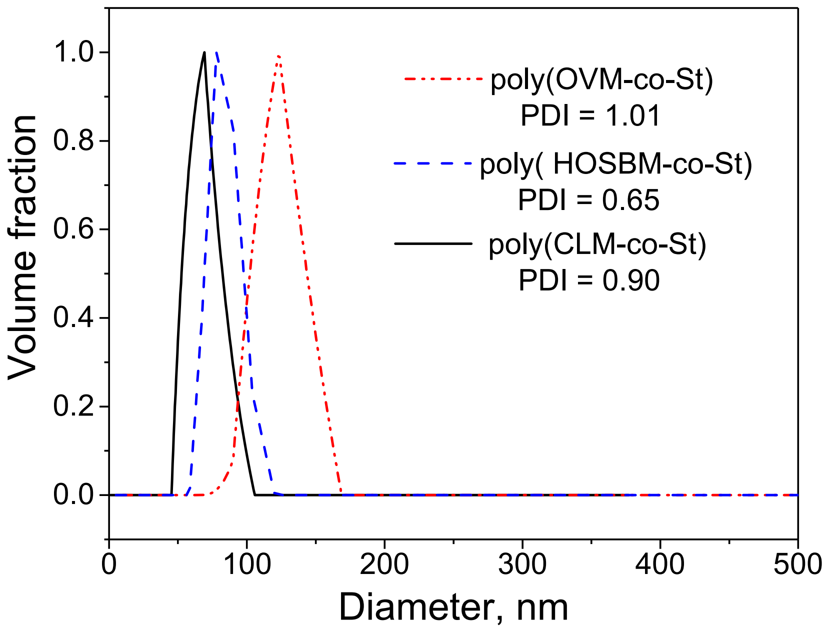

Figure S2. Latex particle size distribution POBM-St 60–40 wt.%.
